# Comparing healthcare needs by language: interpreted Arabic and Somali telehealth calls in two regions of Sweden, 2014–18

**DOI:** 10.1093/eurpub/ckae028

**Published:** 2024-05-22

**Authors:** Leah J Martin, Sharon Kühlmann-Berenzon, Fatima Azerkan, Pär Bjelkmar

**Affiliations:** Public Health Agency of Sweden, Solna, Sweden; Public Health Agency of Sweden, Solna, Sweden; Public Health Agency of Sweden, Solna, Sweden; Public Health Agency of Sweden, Solna, Sweden

## Abstract

**Background:**

Limited language fluency can impede healthcare system navigation. In Sweden, the national telehealth line (Healthcare Guide 1177) offers interpretation in Arabic and Somali. We compared calls by language to identify differences in healthcare use for immigrant populations, focusing on three contact causes: pregnancy; vomiting or nausea in children; and worry/anxiety.

**Methods:**

We conducted a cross-sectional analysis of 3.9 million (*n* = 18 351 Arabic, *n* = 7199 Somali) telehealth calls (2014–18). Using multivariable logistic regression, we investigated associations between language of the call (Arabic, Somali, non-interpreted) and each contact cause. Potential confounders (age, region, year, and additionally for vomiting or nausea, month) and an interaction between age and language were considered.

**Results:**

Compared with non-interpreted calls, interpreted calls were associated with increased odds of being for pregnancy, especially for 19 to 29-year-olds [adjusted odds ratio (aOR) (95% CI) = 4.04 (3.66–4.46) and 4.60 (4.05–5.23), for Arabic and Somali calls, respectively]. Vomiting or nausea showed similar results, with aOR increasing with age: from 0.90 (0.75–1.07) (Arabic, <1 year) to 3.79 (2.86–5.01) (Somali, 5–9 years). In contrast, in unadjusted analyses, Arabic and Somali calls were associated with decreased odds of being for worry/anxiety [OR = 0.47 (0.38–0.58) and 0.34 (0.21–0.50)], respectively, compared with non-interpreted calls.

**Conclusion:**

Our results suggest callers to the interpreted lines may need additional assistance navigating the healthcare system for pregnancy and for vomiting or nausea among children. These findings can inform healthcare services planning for immigrants to Sweden and highlight a novel use of telehealth data as a way to uncover disparities in healthcare use within a multi-linguistic population.

## Introduction

A large and growing proportion of the Swedish population is foreign-born, making up 20% of Sweden’s 2022 population of 10.5 million.^1^ Between 2014 and 2021, over 366 000 individuals sought asylum in Sweden, primarily citizens of Syria (27%), Afghanistan (14%), Iraq (9%) and Eritrea (7%) as well as Somalia (4%).^2^ Many are not fluent in Swedish, which can be a barrier to navigating the country’s healthcare system.

Healthcare in Sweden is universal, largely tax-financed, and operated by the regions and municipalities.^3^ Generally, patients are charged a user fee, which differs by, for example, region, type of visit and entitlement to care.^4^ A number of healthcare services are free, including visits to maternity care and child healthcare centres.^4^ Adult asylum-seekers and undocumented migrants in Sweden have the right to emergency care as well as other forms of care, including maternity and abortion care, while those under 18 years of age have the right to the “same healthcare” as residents.^5^^,^^6^ Language interpretation can be provided but should be planned in advance.^6^

A common entry point to the healthcare system in Sweden is a national telephone health line, Healthcare Guide 1177, which is staffed by nurses and is free to call, other than any telephone charges.^7^ Calls are accepted in a limited number of languages. On the main line, calls can be received in Swedish and English without the need for interpreters and, since 2014, interpreted telephone lines have been offered in Arabic and Somali in three regions of the country with the largest populations: Stockholm, Skåne and Västra Götaland (VGR). The Swedish language line can be challenging for those not fluent in Swedish^8^; however, via interpreted lines, individuals have an opportunity to discuss their health concerns in a language they are comfortable speaking.

Call data from Healthcare Guide 1177 has been investigated for use in national surveillance activities for several diseases in Sweden^9–12^; however, language of the call has not yet been examined. Use of telehealth has been found to vary by ethnicity,^13^ deprivation,^14^^,^^15^^,^^16^ and socioeconomic status,^17^ and, in the US, to be lower for those less proficient in English.^18^ Few studies have, however, compared reasons for calling nursing triage lines like Healthcare Guide 1177 by language of the call. A study in Sweden reported no differences in reasons for calling a nursing triage line by language fluency,^19^ but all the calls were in Swedish. Similarly, a US study found no differences in reasons for calling a nursing triage line by the caller’s need for an interpreter,^20^ but the language of the call was not considered and reasons for calling were not investigated in detail. Others have looked more specifically at language; however, investigations have focused on telephone help lines limited to specific health concerns (e.g. smoking cessation^21^^,^^22^ and rheumatology^23^).

Comparing reasons for calling by language offers a novel use of telehealth call data and can help to identify differences in healthcare, and healthcare information, needs among non-native-speaking immigrant populations compared with their native-language-speaking counterparts. It may also suggest an indication of unmet needs in the regular health care system. The results of this study can be used to improve and expand upon information provided by telehealth services so that they can better respond to the changing needs of the population. Identifying differences in healthcare needs by language is especially important in Sweden, a multicultural and multilingual country that does not routinely collect data on ethnicity or mother tongue.^24^

Our main objective is to quantify and compare interpreted telehealth calls (in Arabic and Somali) to non-interpreted telehealth calls by reason for calling. We focus on calls related to pregnancy; vomiting or nausea among children; and worry/anxiety because these showed some of the largest differences in relative frequency between interpreted and non-interpreted calls in our preliminary analyses. The first two causes were over-represented in the interpreted calls and the latter under-represented.

## Methods

### Telehealth call data

Over four years (24 February 2014 to 9 June 2018) of Healthcare Guide 1177 call data were extracted for two regions: Skåne and VGR (2018 populations: 1 362 164 and 1 709 814, respectively; approximately 30% of the Swedish population).^25^ We compared calls between the non-interpreted line and the two interpreted language lines (Arabic and Somali) by age, sex, region, time period and the reason for calling. Calls that did not result in creation of a medical record were not included; these were more likely to be administrative.

#### Age and sex

Upon calling Healthcare Guide 1177, a caller can enter the Swedish personal identity number for the subject of the call or provide it to the nurse, from which age and sex can be determined. Age and sex can also be provided directly to the nurse. We considered age >111 years an error (based on the oldest person in Sweden at the time of analysis^26^) and set these occurrences to missing.

#### Reasons for calling

The nurses answering Healthcare Guide 1177 calls assess the healthcare needs of the subject of the call using a medical decision support system.^27^ The assessment categorizes the call with respect to one, and only one, reason for calling (‘contact cause’) based on a predefined list. We defined a call for ‘pregnancy’ as one for ‘pregnancy concerns’ or ‘bleeding during pregnancy’ and a call for ‘vomiting or nausea’ in children as one categorized as such in infants, children, or adults as long as the age listed for the call was <10 years. For ‘worry/anxiety’ calls, we included those from subjects aged ≥10 years as we assumed that calls for this reason listing ages <10 years would have likely been for an adult caller, perhaps worried about his/her child (*n* = 445 calls), and we included calls missing age because of the large number of calls in this category.

### Analyses

For each outcome (pregnancy; vomiting or nausea; worry/anxiety), we dichotomized calls as those for (i) the contact cause of interest or (ii) any other contact cause. The exposure variable of interest was language of the call (Arabic, Somali, non-interpreted). In descriptive statistics, we used Wilcoxon rank sum tests to compare continuous variables and χ^2^-tests for categorical variables. We then conducted separate logistic regression analyses for each outcome. For pregnancy, we conducted two separate analyses, the first limited to females 19–39 years of age and the second to calls missing sex and age. We limited the age range in this way because of the low number of interpreted calls for pregnancy among younger (14- to 18-year-olds, *n* = 6) and older (40- to 49-year-olds, *n* = 43) females. Calls for pregnancy missing age and sex were analysed separately to address the larger proportion of interpreted calls missing demographic data. For all three outcomes, we considered age category, region, and year as potential confounders. Additionally, for vomiting or nausea, we considered month as a potential confounder, which was included to account for the seasonality of illnesses, such as acute gastroenteritis caused by norovirus, which often have vomiting and nausea as symptoms. In multivariable analyses, covariates were included if associated with the outcome in bivariable regression at *P* < 0.20. Interactions between language and age were tested using the Likelihood Ratio Test to compare nested models. As the number of interpreted calls for worry/anxiety was low, we did not perform multivariable analysis for this outcome.

Ethical approval for this study was received from the Stockholm Regional Ethics Board (Dnr: 2018/90-31/5). Analyses were conducted in R4.0.3 and SAS 9.4.

## Results

### Language and demographics

Of 3.9 million calls, 25 550 were interpreted [*n* = 18 351 (72%) Arabic and *n* = 7199 (28%) Somali] (table 1). Compared with non-interpreted calls, the median age was lower and the proportion of calls missing age/sex was higher for interpreted calls (*P* < 0.0001) (table 1). Many of the most common reasons for calling were similar among interpreted and non-interpreted calls (Supplementary material). Overall, the three reasons for calling we focused on accounted for 170 045 calls, making up 4.3% of all calls received (14.8% of Somali calls, 8.9% of Arabic calls and 4.3% of non-interpreted calls).

**Table 1:** Characteristics of telehealth calls by language, Skåne and VGR, Sweden, 2014–18

Variable	Value	Language, *n* (%)^a^		
		Non-interpreted (*N* = 3 899 602)	Arabic (*N* = 18 351)	Somali (*N* = 7199)
Age, years	Median (IQR)	27 (7–50)	23 (2–33)	14 (1–29)
Age category, years	<1	231 767 (5.9)	1872 (10.2)	983 (13.7)
	1–4	503 005 (12.9)	3044 (16.6)	1665 (23.1)
	5–9	234 554 (6.0)	1101 (6.0)	388 (5.4)
	10–18	264 971 (6.8)	728 (4.0)	360 (5.0)
	19–29	678 056 (17.4)	3265 (17.8)	1552 (21.1)
	30–39	423 238 (10.9)	2800 (15.3)	1001 (13.9)
	≥40	1 215 544 (31.2)	2351 (12.8)	424 (5.9)
	Missing	348 467 (8.9)	3190 (17.4)	856 (11.9)
Sex	Female	2 068 080 (53.0)	8269 (45.1)	3949 (54.9)
	Male	1 502 530 (38.5)	6374 (34.7)	2238 (31.1)
	Missing	328 992 (8.4)	3708 (20.2)	1012 (14.1)
Region	Skåne	1 291 991 (33.1)	7885 (43.0)	1062 (14.8)
	Västra Götaland (VGR)	2 607 611 (66.9)	10 466 (57.0)	6137 (85.2)
Year	2014	945 833 (24.3)	2939 (16.0)	1429 (19.8)
	2015	959 424 (24.6)	4277 (23.3)	2161 (30.0)
	2016	875 204 (22.4)	5073 (27.6)	1919 (26.7)
	2017	802 895 (20.6)	3626 (19.8)	847 (11.8)
	2018	316 246 (8.1)	2436 (13.3)	843 (11.7)

Notes: All comparisons by language are statistically significantly different at *P* < 0.0001 using Wilcoxon rank sum tests comparing age of non-interpreted calls to each of Arabic- and Somali-language calls and χ^2^-tests comparing the categorical variables by language. IQR, interquartile range.

a Frequency and column percent, by variable, except first row.

### Pregnancy

**Figure 1:**
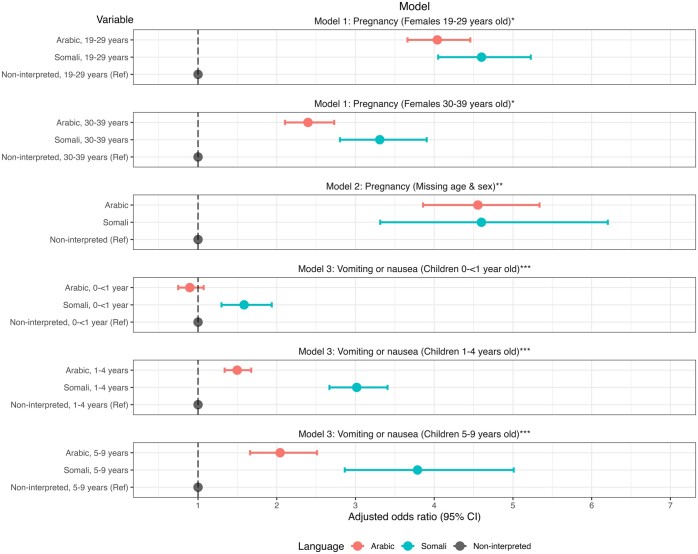
Adjusted odds ratios and 95% confidence intervals (CIs) for each multivariable logistic regression model showing relationships between language and reason for the call, Skåne and VGR, Sweden, 2014–18. Notes: *Model 1 included main effects language, age, year, and region and the interaction between age and language. **Model 2 included main effects language and region. ***Model 3 included main effects language, age, region, year, and month and the interaction between age and language

Among 728 076 calls for females 19–39 years old and among 307 860 calls missing age and sex, 53 941 (7.4%) and 4090 (1.3%), respectively, were for pregnancy. Adjusting for year and region, the odds of the call being for pregnancy were higher for those calling the interpreted lines compared with the non-interpreted line among females 19–39 years of age and varied by age (table 2, figure 1). Among calls for females 19–29 years old, the odds of the call being for pregnancy were 4.04 times higher for Arabic (95% CI = 3.66–4.46) and 4.60 times higher for Somali calls (95% CI = 4.05–5.23) compared with non-interpreted calls. Among calls for females 30–39 years old, similar relationships were seen, but with lower odds ratios (ORs): OR = 2.40 (95% CI = 2.10–2.73) for Arabic and OR = 3.31 (95% CI = 2.80–3.90) for Somali calls (table 2, figure 1). Results for calls missing age and sex were consistent (table 2, figure 1).

**Table 2:** Pregnancy: Unadjusted and adjusted relationships between language and pregnancy-related telehealth calls among females 19–39 years old and calls missing sex and age, Skåne and VGR, Sweden, 2014–18

Calls included	Model	Age group, years	Language	No. calls by contact cause		OR	95% CI		*P*-value
				Pregnancy	Other reasons				
Females 19–39 years old (*N* = 728 076)	Unadjusted	19–39	Arabic	799	3105	3.27	3.03	3.54	<0.0001
			Somali	502	1567	4.07	3.68	4.51	<0.0001
			Non-interpreted (ref)	52 640	669 463	1.00	–	–	–
	Adjusted^a^	19–29	Arabic	522	1739	4.04	3.66	4.46	<0.0001
			Somali	321	927	4.60	4.05	5.23	<0.0001
			Non-interpreted (ref)	31 537	421 769	1.00	–	–	–
		30–39	Arabic	277	1366	2.40	2.10	2.73	<0.0001
			Somali	181	640	3.31	2.80	3.90	<0.0001
			Non-interpreted (ref)	21 103	247 694	1.00	–	–	–
Missing Sex and age (*N* = 307 860)	Unadjusted	–	Arabic	162	2661	4.71	4.00	5.53	<0.0001
			Somali	42	725	4.48	3.27	6.12	<0.0001
			Non-interpreted (ref)	3886	300 384	1.00	–	–	–
	Adjusted^b^	–	Arabic	162	2661	4.55	3.86	5.34	<0.0001
			Somali	42	725	4.60	3.31	6.21	<0.0001
			Non-interpreted (ref)	3886	300 384	1.00	–	–	–

a Multivariable logistic regression model for females 19–39 years old included main effects language, age, region and year and interaction between age and language.

b Multivariable logistic regression model for calls missing sex and age included main effects language and region.

### Vomiting or nausea in children

Among 978 379 calls for children <10 years of age, 66 212 (6.8%) were for vomiting or nausea (table 3). Adjusting for region, month and year, the odds that an interpreted call would be for vomiting or nausea compared with another reason varied by language and age. For Arabic calls, compared with non-interpreted calls in the same age group, no difference was seen for infants <1-year-old but the odds ratio rose to 1.50 (95% CI = 1.34–1.68) for 1- to 4-year-olds and 2.04 (95% CI = 1.66–2.51) for 5- to 9-year-olds (table 3, figure 1). For Somali calls, compared with non-interpreted calls in the same age group, we observed higher odds for infants <1-year-old (OR = 1.59, 95% CI = 1.30–1.94), with the odds increasing to 3.02 (95% CI = 2.67–3.41) for 1- to 4-year-olds and 3.79 (95% CI = 2.86–5.01) for 5- to 9-year-olds (table 3, figure 1).

**Table 3:** Vomiting or nausea: Unadjusted and adjusted relationships between language and telehealth calls related to vomiting or nausea among children <10 years old, Skåne and VGR, Sweden, 2014–18

Model	Age, years	Language	No. calls by contact cause		OR	95% CI		*P*-value
			Vomiting/Nausea	Other				
Unadjusted (*N* = 978 379)	0–9	Arabic	580	5437	1.49	1.36	1.62	<0.0001
		Somali	499	2537	2.74	2.49	3.02	<0.0001
		Non-interpreted (ref)	65 133	904 193	1.00	–	–	–
Adjusted^a^ (*N* = 978 379)	0–<1	Arabic	130	1742	0.90	0.75	1.07	0.23
		Somali	110	873	1.59	1.30	1.94	<0.0001
		Non-interpreted (ref)	17 187	214 580	1.00	–	–	
	1–4	Arabic	349	2695	1.50	1.34	1.68	<0.0001
		Somali	330	1335	3.02	2.67	3.41	<0.0001
		Non-interpreted (ref)	37 676	465 329	1.00	–	–	
	5–9	Arabic	101	1000	2.04	1.66	2.51	<0.0001
		Somali	59	329	3.79	2.86	5.01	<0.0001
		Non-interpreted (ref)	10 270	224 284	1.00	–	–	–

a Multivariable logistic regression model included main effects language, age, region, year, and month and interaction between age and language.

### Worry/anxiety

Of the 2.95 million calls for those ≥10 years of age (or missing age), 45 802 (1.6%) were related to worry/anxiety. Of these, few were made to the interpreted lines (*n* = 92 Arabic and *n* = 22 Somali) and a large proportion (24%) were missing both sex and age. The unadjusted odds of the call being for worry/anxiety was lower for interpreted than non-interpreted calls: OR = 0.47 (95% CI = 0.38–0.58) for Arabic and OR = 0.34 (95% CI = 0.21–0.50) for Somali calls.

## Discussion

This research highlights differences in reasons for calling telehealth services by language in two regions of Sweden. Compared with non-interpreted calls, after adjusting for potential confounding, calls to the Arabic- and Somali-language lines were associated with higher odds of being for pregnancy-related concerns among females (and those missing demographic information) and for vomiting or nausea among children. Overall, these differences suggest a greater need for assistance in accessing information and navigating the healthcare system, specifically for these concerns, among those calling the interpreted lines compared with the non-interpreted line. Furthermore, unadjusted results suggest that interpreted calls were associated with lower odds of being for worry/anxiety compared with non-interpreted calls.

Immigrant women can experience disparities in their prenatal care compared with non-immigrant women.^28^ In our study, this may be reflected by Somali- and Arabic-speaking women seeking proportionately more help for pregnancy concerns, relative to other reasons for calling, compared with women calling the non-interpreted line. Callers to the interpreted lines may need support navigating the Swedish healthcare system, which may be much different from that in their countries of birth. For example, despite maternal health care being offered free-of-charge in Sweden, Somali-born women have been found to be more likely to seek antenatal care later in their pregnancies and make fewer visits than Swedish-born women.^29^ Somali-born parents have described difficulties with antenatal care in Sweden, including issues associated with accessibility, health literacy, communication and stereotyping.^30^ Women born outside Sweden may also lack a circle of family and friends in the country who would normally provide support.^31^ These factors could increase their need for help from a telehealth service that offers unscheduled, quick access to information in their native language and an opportunity to ask questions. Importantly, women who use the interpreted services may have lower levels of education and literacy, and poorer health literacy,^32^ than those using the non-interpreted line, which may increase the inherent value of an interpreted telephone service for this group, especially over print or online resources. Further research is needed to understand how Arabic- and Somali-speaking women use telehealth services for antenatal healthcare and how it can most effectively meet the needs of pregnant women seeking care in languages other than Swedish. Access to telehealth services in one’s own language could be a key source of information and support.

Similar to pregnancy-related calls, the odds of a call for children being for vomiting or nausea were higher on the interpreted lines compared with the non-interpreted line. Although vomiting has many different causes, in Sweden, vomiting during the winter months is often caused by norovirus and referred to as ‘winter vomiting disease’. Swedish-born parents, or those who have lived in the country longer, are likely familiar with this illness and may not seek care as they feel they know how to respond. However, for parents from other countries, a vomiting child could be more concerning. Furthermore, callers to the interpreted lines may have lower levels of education and health literacy^32^ and thus may not fully understand printed resources and be uncertain about how to treat a vomiting child or when to seek care. Therefore, although the disease burden may be similar in each of these two groups, the need for healthcare information, and telehealth assistance in particular, may be greater for parents from other countries calling the interpreted lines.

Overall, ‘worry/anxiety’ was not a common reason for calling Healthcare Guide 1177, but it was even less common on the interpreted lines. Worry and anxiety are considered differently across cultures and these differences need to be considered when interpreting our results.^33^ The lower unadjusted odds of seeking assistance via telehealth for mental health concerns on the interpreted lines should not be considered an indication that Arabic or Somali speakers experience fewer mental health issues. Rather, it suggests that these groups are not using this service for these reasons.

Although the telephone number ‘1177’ is nationally available and well-known in Sweden, during the study period, the telephone numbers for the interpreted lines were not as easy to remember and were not advertised to the same extent, nor were the services available 24 hours a day; interpreted services were also only available in three regions (Stockholm, VGR and Skåne) together representing approximately 53% of the Swedish population.^25^ This highlights the more limited coverage of interpreted vs. non-interpreted Healthcare Guide 1177 services in Sweden. At the time of writing, interpretation is offered in additional languages, including Persian (in VGR and Stockholm)^7^^,^^34^ and Russian and Tigrinya (in Stockholm).^7^ In Skåne and VGR, interpreted services are now accessible directly via the commonly known 1177 telephone number. Nevertheless, geographic coverage and hours of availability remain more limited for interpreted calls.^7^^,^^34^

To our knowledge, this study presents a novel use of telehealth call data and is one of the first studies to examine reasons for calling telehealth by language of the call using a large, population-based telehealth call database. Some studies have, however, compared telehealth call characteristics by the caller’s level of fluency or need for an interpreter. For example, in the United States, calls to a nursing triage line from patients who needed an interpreter lasted longer and were more likely to be made by a surrogate and be rated as more urgent compared with calls from patients not needing an interpreter; however, no differences in the chief complaints underlying the calls or the number of calls per patient were found.^20^ A previous study in Sweden examined a sample of 800 telehealth calls (recorded in 2004–05) conducted in Swedish and found that the most common reasons for calling were similar by Swedish language fluency.^19^ In Norway, non-fluent Norwegian speakers calling a casualty clinic telephone consultation line were less likely to trust the nurse and less likely to report receiving relevant answers to their questions compared with fluent speakers^35^; however, reasons for calling were not compared. Although our results are specific to Sweden, our finding that reasons for calling telehealth differ by language is relevant to consider in other countries, and these differences could be useful as indicators of differing healthcare needs among immigrant communities. Nevertheless, given the lack of evidence in this area, more international research is needed.

This study has a number of potential limitations. First, these are secondary data with possible data entry errors. Age and sex were likely inaccurately entered for some calls and, in some instances, demographics may represent the caller rather than the subject of the call. Providing a personal identity number automatically gives the age and sex; therefore, groups lacking identity numbers (e.g. asylum-seekers, undocumented migrants, recent immigrants, or non-residents) may be more likely to be missing demographic information. However, we designed the analyses to minimize the effect of missingness. Second, we cannot explore how other factors (e.g. education level, time in Sweden, asylum-seeker status, language proficiency) might affect our results. Third, the unit of analysis was the call, not the individual; therefore, we could not adjust for the frequency with which each individual called. If individuals calling the interpreted lines for pregnancy or for vomiting or nausea were more likely to call repeatedly for the same reason than those on the non-interpreted line, this would bias the results away from the null. Finally, the non-interpreted line is used by a mixture of callers who can speak Swedish and/or English, likely with a range of fluency. Therefore, it conceals a proportion of callers who, similar to the interpreted lines, may be unfamiliar with the Swedish healthcare system and illnesses common in Sweden; these individuals may be similar, in some respects, to those calling the interpreted lines. This remains an area for future analysis.

### Recommendations

More work is needed to understand immigrant health and healthcare services in countries like Sweden for people who cannot speak the dominant language fluently enough to receive effective healthcare services without the assistance of interpretation. For example, qualitative research could explore and compare the knowledge, attitudes, and practices of asylum-seekers and other immigrants regarding the healthcare system in Sweden. Although we selected three contact causes that differed by language, several others warrant attention (e.g. dental problems). Additional languages are now offered by the interpretation services that could be studied, as well as data from other regions in Sweden. Furthermore, this type of investigation should be continued with data during the COVID-19 pandemic and forward. Moreover, these types of public health surveillance data should be considered as potential data sources to understand differences in healthcare needs in other countries.

Healthcare Guide 1177 is a first point of contact for healthcare in Sweden. The convenience of calling for advice rather than seeking care in person is ideal for someone unfamiliar with one’s environment (e.g. immigrants) and those who have difficulty leaving the house (e.g. parents of young children); this latter group is especially important to consider since a high proportion of calls is for young children. These services should be expanded to ensure all residents of Sweden can access telephone healthcare advice in a language in which they are fluent.

## Conclusion

Although many of the main reasons for calling telehealth were similar for the non-interpreted and interpreted lines, compared with non-interpreted calls, calls in Arabic and Somali were associated with higher odds of being for pregnancy and, among children, for vomiting or nausea. These differences could be interpreted as indicators of differing healthcare needs among immigrant communities. Our results could inform planning and development of healthcare services for immigrants in Sweden.

## Supplementary Material

ckae028_Supplementary_Data

## Data Availability

The data underlying this article cannot be shared publicly as they contain confidential data from 1177. Key points Few studies have examined reasons for calling telehealth by language of the call using a population-based telehealth call database. Our results showed that, compared with non-interpreted calls (mainly in Swedish), interpreted calls in Arabic and Somali had higher odds of being for pregnancy-related concerns and, among children, for vomiting or nausea. This study highlights a novel use of telehealth call data as a way to uncover disparities in healthcare use among a multi-linguistic population; this has the potential to be highly transferable to other countries. Our findings suggest that Arabic and Somali speakers have unmet healthcare needs within the regular healthcare system; these gaps should be considered in healthcare services planning for immigrant populations.
