# Supplementary material for: Comparing healthcare needs by language: interpreted Arabic and Somali telehealth calls in two regions of Sweden, 2014–18
Source: Eur J Public Health. 2024 May 22;34(3):537–43. doi: 10.1093/eurpub/ckae028 (PMC11161146; doi:10.1093/eurpub/ckae028)
Supplement: ckae028_Supplementary_Data [file ckae028_supplementary_data.docx]

**Supplemental Materials**

**Title:** Comparing healthcare needs by language: Interpreted Arabic and Somali telehealth calls in two regions of Sweden, 2014-2018

**Authors:** Leah J. Martin, Sharon Kühlmann-Berenzon, Fatima Azerkan, Pär Bjelkmar

**Affiliations:** Public Health Agency of Sweden

**Table S1. Swedish text and corresponding English translation for contact causes (reasons for calling) considered in analyses**

| **English** | **Swedish** |
| --- | --- |
| Pregnancy concerns | “graviditetsbesvär” |
| Bleeding during pregnancy | “blödning under graviditet” |
| Vomiting or nausea | “kräkning eller illamående” |
| Worry, anxiety | “oro, ångest” |

**Table S2. Most common contact causes by language and sex**

| **Sex** | **Language** | | |
| --- | --- | --- | --- |
|  | **Arabic** | **Somali** | **Non-interpreted** |
| Female | 1. Fever - child (7%) 2. Pregnancy concerns (7%) 3. Administrative (6%) 4. Abdominal pain - adult (5%) | 1. Pregnancy concerns (10%) 2. Fever - child (6%) 3. Vomiting or nausea - child (5%) 4. Abdominal pain - adult (5%) | 1. Abdominal pain -adult (6%) 2. Medication question (4%) 3. Fever - child (4%) 4. Administrative (4%) |
| Male | 1. Fever - child (10%) 2. Administrative (7%) 3. Vomiting or nausea – child (4%) 4. Rash - child (3%) | 1. Fever - child (13%) 2. Vomiting or nausea – child (9%) 3. Administrative (4%) 4. Rash - child (4%) | 1. Fever - child (6%) 2. Administrative (4%) 3. Abdominal pain -adult (3%) 4. General medical information (3%) |
| Missing sex | 1. Administrative (19%) 2. General medical information (8%) 3. Fever - children (5%) 4. Pregnancy concerns (4%) | 1. Administrative (25%) 2. General medical information (8%) 3. Medication question (5%) 4. Pregnancy concerns (4%) | 1. General medical information (17%) 2. Administrative (14%) 3. Medication question (10%) 4. Worry, anxiety (3%) |
| **Total** | 1. Administrative (9%) 2. Fever – child (7%) 3. General medical information (4%) 4. Pregnancy concerns (4%) | 1. Fever – child (8%) 2. Administrative (7%) 3. Vomiting or nausea – child (6%) 4. Pregnancy concerns (6%) | 1. Fever – child (5%) 2. Administrative (5%) 3. Abdominal pain – adult (5%) 4. General medical information (4%) |
